# Mining Candidate Genes for Leaf Angle in *Brassica napus* L. by Combining QTL Mapping and RNA Sequencing Analysis

**DOI:** 10.3390/ijms25179325

**Published:** 2024-08-28

**Authors:** Aoyi Peng, Shuyu Li, Yuwen Wang, Fengjie Cheng, Jun Chen, Xiaoxiao Zheng, Jie Xiong, Ge Ding, Bingchao Zhang, Wen Zhai, Laiqiang Song, Wenliang Wei, Lunlin Chen

**Affiliations:** 1 College of Agriculture, Yangtze University, Jingzhou 434025, China; 13663996452@163.com (A.P.); 13872234221@163.com (F.C.); 2 Jiangxi Province Key Laboratory of Oil Crops Genetic Improvement (2024SSY04031), Nanchang 330200, China; lishuyu0104@163.com (S.L.); m18175426086@163.com (X.Z.); ixiongjie@163.com (J.X.); gedingjxaas@163.com (G.D.); zwzpzbc@163.com (B.Z.); songlq168@163.com (L.S.); 3 Crop Institute, Jiangxi Academy of Agricultural Sciences, Nanchang 330200, China; 4 Fuzhou Teachers’ College, East China University of Technology, Fuzhou 344000, China; 201860169@ecut.edu.cn (J.C.); zhaiwen1105@163.com (W.Z.)

**Keywords:** rapeseed, leaf angle, QTL mapping, transcriptome, cell wall

## Abstract

Leaf angle (LA) is an important trait of plant architecture, and individuals with narrow LA can better capture canopy light under high-density planting, which is beneficial for increasing the overall yield per unit area. To study the genetic basis and molecular regulation mechanism of leaf angle in rapeseed, we carried out a series of experiments. Quantitative trait loci (QTL) mapping was performed using the RIL population, and seven QTLs were identified. Transcriptome analysis showed that the cell wall formation/biogenesis processes and biosynthesis/metabolism of cell wall components were the most enrichment classes. Most differentially expressed genes (DEGs) involved in the synthesis of lignin, xylan, and cellulose showed down-regulated expression in narrow leaf material. Microscopic analysis suggested that the cell size affected by the cell wall in the junction area of the stem and petiole was the main factor in leaf petiole angle (LPA) differences. Combining QTL mapping and RNA sequencing, five promising candidate genes *BnaA01G0125600ZS*, *BnaA01G0135700ZS*, *BnaA01G0154600ZS*, *BnaA10G0154200ZS*, and *BnaC03G0294200ZS* were identified in rapeseed, and most of them were involved in cell wall biogenesis and the synthesis/metabolism of cell wall components. The results of QTL, transcriptome analysis, and cytological analysis were highly consistent, collectively revealing that genes related to cell wall function played a crucial role in regulating the LA trait in rapeseed. The study provides further insights into LA traits, and the discovery of new QTLs and candidate genes is highly beneficial for genetic improvement.

## 1. Introduction

High-density planting is an important measure to increase the yield of rapeseed per unit area [1,2,3,4,5]. As the planting density of rapeseed in the Yangtze River Basin of China was improved from 150,000–300,000∙hm^−2^ to 450,000–600,000∙hm^−2^, the yield increased by 20% and reached 2400–2700 kg∙hm^−2^ [6]. Nevertheless, high-density planting resulted in the shade-avoidance response of plants, increasing competition for light, water, and fertilizer and decreasing photosynthetic utilization rate, which ultimately reduced yield. For the winter rapeseed genotype, the optimal planting density for the highest yield was 300,000–600,000 hm^−2^, and exceeding this planting density would decrease the survival rate of plants [7]. Plant architecture improvement is critical to maximizing crop yield per unit area [8].

In high-density planting, rapeseed varieties with a narrow leaf angle can reduce the shading among leaves, increase light penetration through the leaf canopy, achieve more uniform light capture, and promote more accumulation of photosynthates during the vegetative growth period [9]. LA can be divided into LPA and the leaf inclination angle (LIA). The angle between the main stem and the leaf petiole is defined as LPA, and the LIA refers to the angle of blade inclination [10]. Vertically inclined leaves are conducive to light penetration into the lower canopy and maximize the photosynthesis of plant canopy structure [11].

Many studies on LA were carried out on various crops, such as maize, tomato, rice, and so on [9,12,13,14,15]. Some genes involved in regulating leaf angle have been identified. *OsSLA1*, a leucine-rich repeat receptor-like kinase, was engaged in leaf angle development of rice by regulating the size of adaxial cells and vascular bundles in the leaf node. *ILI1*, *BU1*, and *BUL1* positively regulated BR signal transduction, and their loss of function or inhibition resulted in a narrower leaf angle in rice, while overexpression resulted in a wider LA [16,17,18]. *OsCKX3* negatively regulated leaf angle, as rice *osckx3* mutants exhibit a smaller leaf angle [19]. *GmILPA1* could control the LPA to change the structure of soybean plants. The *Gmilpa1* mutant had a larger petiole angle and a shorter petiole than the wild-type soybean [20]. *GhAPC8* regulated cotton’s leaf blade angle (LBA) by affecting the accumulation and signal transduction of various plant hormones. *GhAPC8* played a role in the regulation of LA by regulating the accumulation of various plant hormones (such as IAA, BR, and GA) [21]. *LsNRL4* encoded the NPH3/RPT2-like (NRL) protein, contributing to chloroplast development and leaf angle reduction in lettuce [22]. Recently, the “smart canopy” gene *lac1*, which regulates the LA trait, was identified in maize, and large-scale field trials showed that *lac1* can enhance maize yields under high-density conditions. This important study also suggests that research on LA traits in rapeseed is of great significance [23].

While studies on LA traits in other important crops are comprehensive, covering both gene cloning and molecular regulatory mechanisms, research on LA in rapeseed remains insufficient. Several significantly associated loci were identified by GWAS, and candidate genes including *IAA7*, *PIN3*, and *BZR1* were speculated to regulate LA in rapeseed. The understanding of the genetic mechanism and cytological mechanism responsible for LA in rapeseed is still poor, which is a constraint to our in-depth understanding and genetic improvement of rapeseed high-density planting. In this study, we carried out QTL mapping using the RIL population constructed by parents (‘M352’ × ‘M201’) with significant differences in leaf angles of rapeseed. Four LPA (*qLPAA01*, *qLPAA10.a*, *qLPAA10.b*, and *qLPAC03*) and three LIA (*qLIAA06*, *qLIAC03.a*, and *qLIAC03.b*) QTLs were detected in two environments. Furthermore, combined with the transcriptome results, five candidate genes (*BnaA01G0125600ZS*, *BnaA01G0135700ZS*, *BnaA01G0154600ZS*, *BnaA10G0154200ZS*, and *BnaC03G0294200ZS*) controlling the leaf angle were identified. Developing molecular markers for the above genetic loci or target gene editing could improve the LA trait in rapeseed, making it easier to achieve high yields under high-density planting conditions. The study helps us better understand the genetic basis of the LA trait, which is of great significance for its genetic improvement in rapeseed.

## 2. Results

### 2.1. Phenotypic Performance of RIL Population for LA Trait

**Figure 1:**
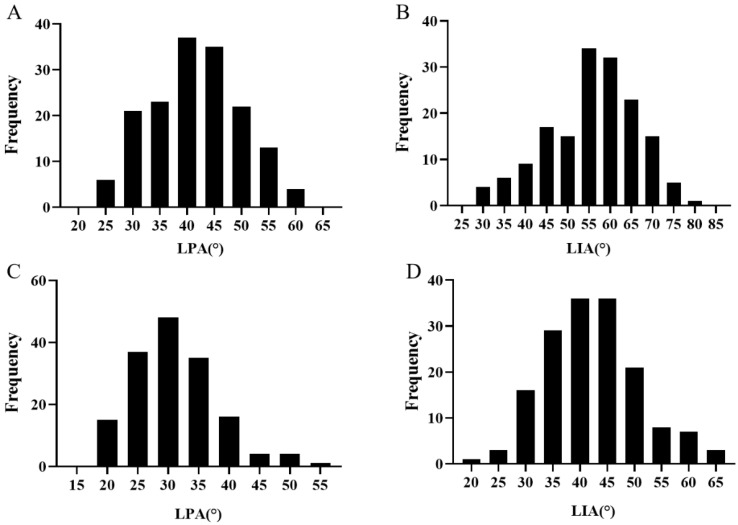
Leaf angle frequency distribution of RIL population in the two environments. (**A**) LPA frequency distribution of RIL population in Gansu. (**B**) LIA frequency distribution of RIL population in Gansu. (**C**) LPA frequency distribution of RIL population in Nanchang. (**D**) LIA frequency distribution of RIL population in Nanchang.

The LPA and LIA of ‘M352’ were both significantly wider than those of ‘M201’ in the two investigated environments (*p* < 0.05) (Table 1). Extensive phenotypic variations for LA of LPA and LIA were observed in the 192 individuals of the RIL population that were grown in two environments (2022 in Nanchang and Gansu) (Figure 1). Across the two environments, LPA varied from 18.44° to 61.69° and LIA varied from 21.31° to 81.04°. As shown in Figure 1, the phenotypic data of LPA and LIA showed an obvious normal distribution (*p* < 0.05), indicating it is suitable for QTL mapping.

### 2.2. QTLs Mapping for LA Trait 

Phenotypic data collected in Gansu and Nanchang were used for QTL analysis. A total of seven QTLs for LA were identified in different environments, among which four loci and three loci were LPA and LIA QTLs, respectively (Table 2). They were distributed on A01 (*qLPAA01*), A06 (*qLIAA06*), A10 (*qLPAA10.a*, *qLPAA10.b*), and C03 (*qLPAC03*, *qLIAC03.a*, *qLIAC03.b*) linkage groups. There were 646 and 234 *B. napus* genes located at the loci of LPA and LIA (Supplementary Table S1). These identified QTLs for LPA and LIA explained 0.44–11.29% and 0.87–17.49% of the phenotypic variance, respectively. 

In this study, most of the identified QTLs were minor effect loci, except for *qLPAC03.a* and *qLIAC03*. In fact, *qLPAC03* and *qLIAC03.a* were the same loci, which simultaneously regulated LPA and LIA traits, with a phenotypic contribution rate of over 10%.

### 2.3. Transcriptome Analysis of LPA

To explore the possible molecular mechanism of regulating LPA in extreme lines R009 and R036, transcriptome sequencing was performed using the tissue of the connection area between the petiole and the stem (Supplementary Figure S1). There were three biological replicates, and a high consistency between different replicates implied the accuracy of RNA-seq (Supplementary Figure S2). Principal component analysis (PCA) was performed on all sequenced genes, and it was observed that R036 separated significantly from R009, indicating that the distribution of R009 and R036 was very different (Supplementary Figure S3).

Each sample produced an average of 62,809,424 bp raw reads, and the average Q30 and GC content of the original RNA-seq data reached 96.66% and 46.12%, respectively (Supplementary Table S2). Each sample produced about 54–76 million clean reads, of which about 93.09% of clean reads could be aligned to ZS11, with about 87.22% of them being uniquely mapped (Supplementary Table S2). In total, 12,555 differentially expressed genes (DEGs; |log2FoldChange| ≥ 1 and *p*-adjust < 0.05) were identified, of which 4736 (37.72%) were up-regulated and 7819 (62.28%) were down-regulated (Supplementary Figure S4).

**Figure 2:**
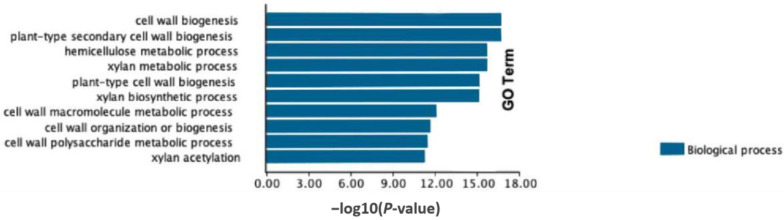
GO enrichment analysis of DEGs between R036 and R009.

These DEGs were used for enrichment analysis with TBtools software (version 2.096) [24]. Through the KEGG enrichment analysis, 120 KEGG enrichment pathways were found, but only the Sulfur metabolism reached a significant level of enrichment (Supplementary Figure S5 and Supplementary Table S3). Sulfur is an essential component of many biomolecules, which may play an important role in the regulation of leaf angle. Among the ten most GO-enriched classes, six were cell wall growth/biogenesis-related biological processes, such as cell wall biogenesis, plant-type secondary cell wall biogenesis, plant-type cell wall biogenesis, and the cell wall macromolecule metabolic process (Figure 2). Furthermore, the remaining GO enrichment classes were related to the biosynthesis and metabolism of essential cell wall components, namely the hemicellulose metabolic process, xylan metabolic process, xylan biosynthetic process, and xylan acetylation (Figure 2). The results indicated the vital role of cell wall activity in regulating LPA traits. 

**Figure 3:**
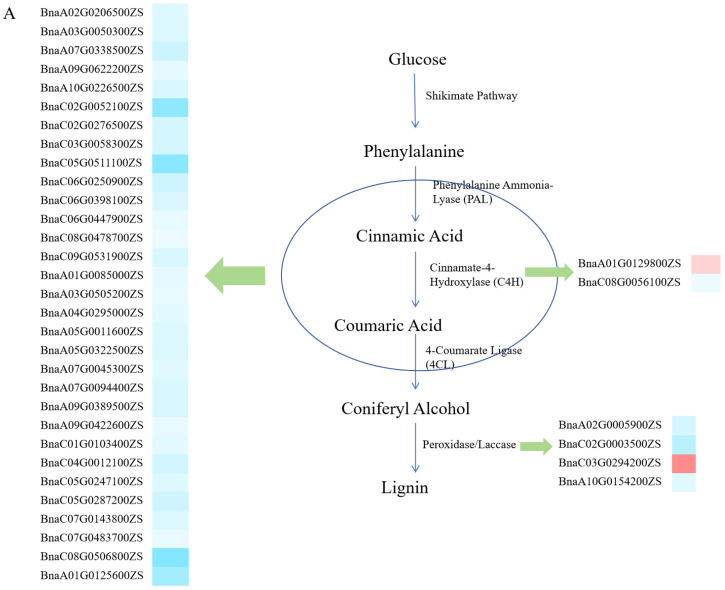

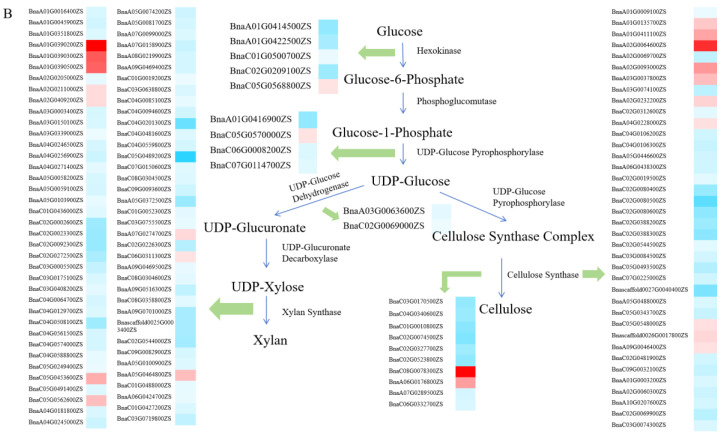
Synthetic pathway of main components in cell wall. (**A**) DEG expression in lignin synthesis. (**B**) DEG expression in xylan and cellulose synthesis.

Given that almost all of the most GO-enriched classes are associated with cell wall biogenesis and the synthesis of the main component in the cell wall, we extracted all the DEGs involved in these biological processes. The functions of these DEGs in the synthesis of lignin, xylan, and cellulose were analyzed. Figure 3 showed that almost all detected DEGs (94.6%) in the lignin synthesis pathway were down-regulated in narrow LPA material R036, except *BnaA01G0129800ZS* and *BnaC03G0294200ZS*. A similar pattern was observed in the synthetic process of xylan and cellulose, that is, most DEGs were down-regulated in narrow leaf material. The results showed that the biosynthesis of cell wall components lignin, xylan, and cellulose was inhibited in R036 compared with R009. The reduction in these key components’ synthesis of the cell wall was likely to lead to the reduction in the cell wall size, which directly affected the size of plant cells. As the cell wall plays an important role in regulating the morphology and size of plant cells, we assume that the morphology or size of cells in key areas may be the reason for differences in LPA between R009 and R036.

### 2.4. The Anatomical Structure of Stem 

**Figure 4:**
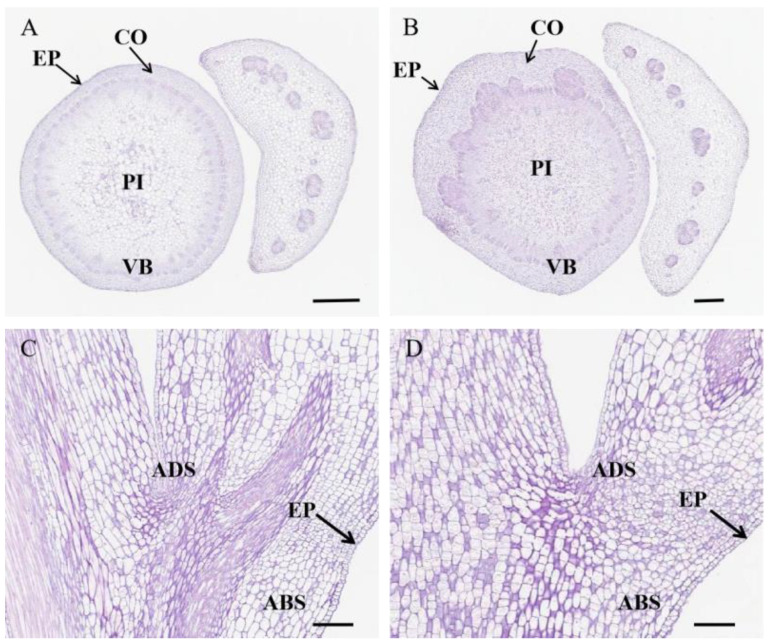
The anatomical structure of the junction between the main stem and leaf petiole. (**A**) The transverse section of the junction tissue of the R036 plant. Scale bars, 0.5 mm. (**B**) The transverse section of the junction tissue of the R009 plant. Scale bars, 0.5 mm. (**C**) The longitudinal section of the junction tissue of the R036 plants. Scale bars, 160 μm. (**D**) The longitudinal section of the junction tissue of the R009 plant. Scale bars, 160 μm. Abbreviations: EP: epidermis CO: cortex PI: pith VB: vascular bundle P: phloem VC: vascular cambium ABS: abaxial side; ADS: adaxial side.

The connection part between the petiole and stem was the target area for paraffin sections. As illustrated in Figure 4, it comprised the epidermis, cortex, vascular column, and pith (Figure 4A,B). The cell diameter and surface area of R009 were 62.3% and 170.6% larger than those of R036, respectively (Figure 4C,D and Table 3). The larger cells in the junction area of the stem and petiole made the petiole further separated from the stem, resulting in a wider leaf petiole angle of R009. Microscopic analysis showed that the cell size in the junction area was the main factor affecting LPA, which is highly consistent with RNA-seq results. 

### 2.5. Combined Analysis of QTL and DEGs

**Figure 5:**
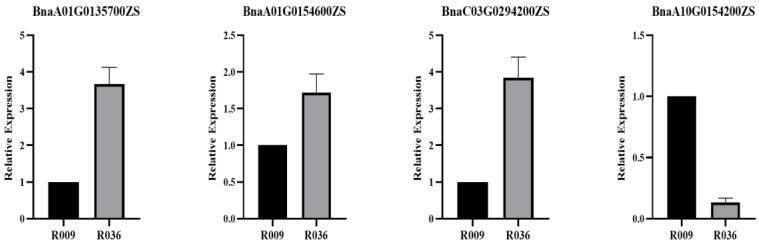
Relative expression of candidate genes in the R036 and R009 by qPCR.

Combined with QTL results and RNA-seq analysis, a total of 81 DEGs were found in QTL regions (Supplementary Table S4). Referring to gene annotation information, five DEGs were regarded to play an important role in regulating LPA. The expression levels of candidate genes in R009 and R036 were identified by qPCR. The qPCR results were consistent with RNA-Seq (Figure 5). In R036, *BnaA01G0135700ZS*, *BnaA01G0154600ZS*, and *BnaC03G0294200ZS* were up-regulated, *BnaA10G0154200ZS* was down-regulated, and *BnaA01G0125600ZS* was not expressed. Five DEGs were distributed in the regions of *qLPAA01* (*BnaA01G0125600ZS*, *BnaA01G0135700ZS*, *and BnaA01G0154600ZS*), *qLPAA10.a* (*BnaA10G0154200ZS*), and *qLPAC03* (*BnaC03G0294200ZS*). *BnaA01G0154600ZS* was homologous to *AT4G25960*, which played a significant role in auxin transport. The *Arabidopsis* homologous genes of *BnaA01G0125600ZS* and *BnaA10G0154200ZS* regulated lignin biosynthesis in secondary cell wall formation or cell wall biosynthesis. *BnaA01G0135700ZS*, homologous to *AT4G24000*, was cellulose synthase. *BnaC03G0294200ZS* was homologous to *AT4G08770*, which is related to cell wall formation (Table 4).

## 3. Discussion

Increasing the planting density is the development trend of rapeseed in the future, which means more population per unit area to increase the total yield. However, high-density planting also brings some challenges, such as insufficient light, poor ventilation, and nutrient competition. Leaf angle trait plays a key role in solving these problems. Rapeseed varieties with smaller leaf angles could reduce shading between leaves, help the plants obtain enough light energy, increase the ventilation between plants, decrease the retention of moisture, and reduce the incidence of pests and diseases [9]. Therefore, the study on leaf angle traits is of great importance in solving the problems brought by high-density planting and increasing the overall yield.

### 3.1. Novel Loci Identified for LA in Rapeseed

Due to the importance of the LA trait in high-density planting, it is essential to conduct genetic research in rapeseed. In *Brassica napus* L., some loci or candidate genes of LPA have been found by QTL mapping or GWAS. There are only two reports on the LA trait of rapeseed, one of which detected 45 significantly associated loci through GWAS and identified several promising candidate genes regulating LPA, including *PIN3*, *IAA7*, and *BZR1* orthologous genes by integrating GWAS and RNA-seq analyses. In another study, a significant GWAS signal on chromosome A03 including a candidate gene *BnaA03g10430D* was identified, *BnaA03g10430D* encoded on an auxin efflux carrier, which could be involved in polar auxin transport, and regulating LPA in rapeseed [25]. Here, we detected six novel loci for the LA trait by QTL mapping. Among these loci, the only major-effect loci *qLIAC03* simultaneously regulated LPA and LIA traits. Developing molecular markers for these identified loci and applying them to breeding is an efficient way to improve LA in rapeseed varieties.

### 3.2. Characteristics of DEGs and Cytological Analysis

Among the two sub-traits of rapeseed LA, LPA is relatively simple. In contrast, LIA is more complex because, in addition to being influenced by LPA, LIA is significantly affected by leaf shape and weight. These multiple factors together determine the LIA trait. Given the complexity of the LIA trait, we chose to analyze extreme LPA materials in cytological and transcriptome studies.

There are usually several cytological reasons for morphological differences in specific plant organs or tissues: the number and arrangement of cells and the cell morphology. For example, Li et al. found that the seed weight of rapeseed was decided by its size mainly via cell number [26]. In cowpeas, cell number was a critical factor in the pod length variations, and the long-pod phenotype of Zhijiang282 was primarily due to more cell proliferation rather than cell elongation/enlargement [27]. Our cytological result showed that the cell size in the ADS area of R009 (wide LPA) was significantly larger than that of R036 (narrow LPA), and cell morphology played a critical role in regulating the LPA trait in rapeseed.

All of the most GO-enriched classes of DEGs were related to cell wall activities. The first and second most enriched categories were cell wall biogenesis and plant-type secondary cell wall biogenesis, respectively, and both of them regulated the formation and strengthening of the cell wall, directly affecting cell shape and size. The structure and substance composition (such as polysaccharides, proteins, and aromatic substances) of the cell wall decide the characteristics of individual cells in plants [28]. On this basis, the plant wall functions as the decisive factor of plant morphology [28]. In our study, four of the ten most enrichment classes were biosynthesis/metabolism/acetylation processes of xylan and hemicellulose. Xylan is a specific and significant type of hemicellulose. Xylan acetylation affected its physical properties, such as cross-linking and solubility. The synthesis and metabolism process of xylan decided the composition and function of the cell wall, thereby influencing cell morphology and size.

Through detailed analysis of the lignin synthetic process, we found that DEGs homologous to NAC and MYB transcription factors played a crucial role in lignin synthesis by regulating the enzyme activity of Phenylalanine Ammonia-Lyase (PAL), Cinnamate-4-Hydroxylase (C4H), and 4-Coumarate Ligase (4CL) (Figure 3A) [29]. These transcription factors showed a trend of down-regulated expression in R036. The MYB transcription factor promoted cell elongation on the adaxial side of the leaf joint to show leaf angle tilt [30]. This is consistent with our findings that MYB expression is lower and that plants show more upright leaves. Most of the DEGs involved in the synthetic process of xylan and cellulose belonged to TBL, IRX, and CESA gene families [31,32,33]. TBL proteins were associated with the acetylation of cell wall polysaccharides, which is a critical regulatory step for the structure and function of these polysaccharides [31]. Some IRX proteins, such as *IRX9*, *IRX10*, and *IRX14*, were involved in xylan synthesis through a Golgi-localized complex termed the xylan synthase complex (XSC) [32]. The CESA gene family-encoded cellulose synthase was directly involved in the cellulose synthetic process and regulated the generation/arrangement of cellulose microfibrils [33]. Many members of the CESA gene family played various roles in the synthesis of cellulose [33]. Furthermore, some identified DEGs in cellulose synthesis were homologous to *FRA1*. The regulation of *FRA1* on the level of the cellulose synthase-microtubule uncoupling (CMU) protein and microtubule localization provides a mechanism for stabilizing the deposition sites of cellulose and matrix polysaccharides [34].

At the molecular level, RNA-seq results proved that genes affecting cell wall biogenesis and the synthesis/metabolism of crucial substances were involved in the regulation of LPA traits.

### 3.3. Potential Regulatory Model for LPA and Identification of Candidate Genes in Brassica napus *L.*

**Figure 6:**
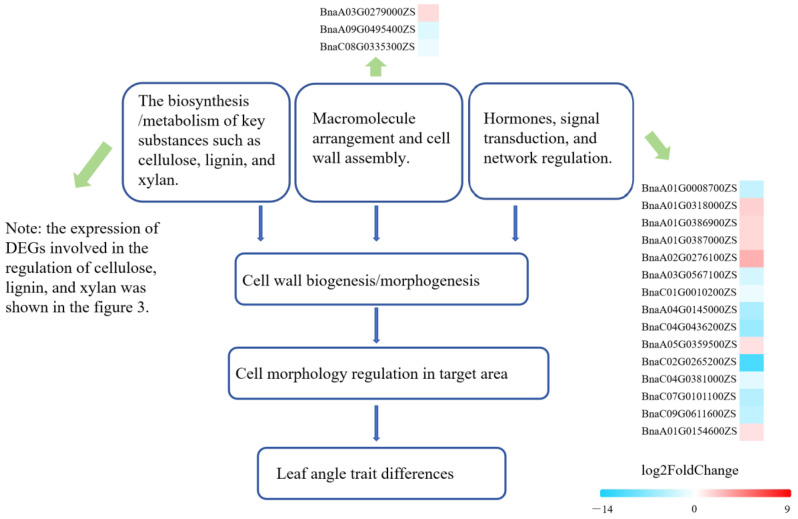
Potential regulatory model for LPA in *Brassica napus* L.

Many factors influenced the biogenesis and morphological characteristics of plant cell walls. These factors can be grouped into three categories: (1) the biosynthesis/metabolism of key substances such as cellulose, lignin, and xylan [35,36]; (2) macromolecule arrangement and cell wall assembly; and (3) macromolecule arrangement and cell wall assembly (Figure 6). Due to the key part of cell wall morphology and physical characteristics in the regulation of cell morphology, these factors also determined the morphological characteristics of plant cells to a large extent. As found in this study, the inhibition of lignin, xylan, and cellulose synthesis made cell size smaller in specific parts of the plant, resulting in narrow LPA in rapeseed. In addition, the number of DEGs identified in the biosynthesis/metabolism of key substances was much more than that in the other two factors, and the candidate genes identified by a meta-analysis of QTL and RNA-seq results also affected the LPA traits by regulating the synthesis/metabolism process of the main components of the cell wall. In the study of rice, there is a consistent view with this study: the number of thick-walled tissue cells in leaf internodes will affect leaf tilt [37]. In tomatoes, *SlARF11* regulates leaf angles by altering cell proliferation or elongation on the adaxial side of the petiole base and IAA and BR [13].

For instance, *BnaC03G0294200ZS* was located in the region of major QTL *qLPAC03*, which was homologous to *AT4G08770* (*AtPrx37*). The plants overexpressing *AtPrx37* showed an increase in esterified phenols related to their cell wall and it was speculated that *AtPrx37* was related to lignin deposition [38]. Lignin was the main regulator of cell wall formation, and peroxidase and laccase played an important role in the synthesis of lignin [35,39,40]. Peroxidase-related candidate genes have been previously mentioned, and another candidate gene, *Laccase 17* (*BnaA10G0154200ZS*), was involved in the deposition of lignin in fibers [39]. Many studies have reported that MYB transcription factors affect the formation of secondary cell walls by regulating the synthesis of lignin and cellulose [41,42]. One of the candidate genes *BnaA01G0125600ZS* belonged to the MYB family, and its homologous gene was *AT4G22680* (*MYB85*). In rice, *OsMYB7* could inhibit the biosynthesis of lignin and increase cellulose content to regulate the formation of the cell wall [30]. *OsMYB7*-overexpressing mutants had wider leaf angles, which made the leaves more inclined mainly by reducing the level of free auxin, increasing the thickening of the hard cell wall, and promoting the cell elongation of the adaxial side of the leaf joint [30]. The *CSLG2*, a homologous gene of *BnaA01G0135700ZS*, was cellulose synthase. *CSLG2* polymerized the hemicellulose skeleton of the plant cell wall as a catalytic enzyme to synthesize non-cellulosic polysaccharides and promote cell wall biogenesis [43]. *BnaA01G0154600ZS* was homologous to *AT4G25960* (*ABCB2*), which had an important contribution to auxin transport [44]. Auxin could promote cell wall expansion by the acidification of the cell wall and the activation of structural proteins and enzymes [45]. Arabidopsis *ABCB19* mutants exhibited erect leaf angles by directly regulating auxin biosynthesis and transport processes [46]. Similarly, in cucumber, *ABCB19* regulated auxin transport and led to more erect and compact leaf architecture [47].

## 4. Materials and Methods

### 4.1. Plant Material and Growth Conditions

A total of 192 lines from the RIL population, together with their parents, were planted in March 2022 at Zhangye City, Gansu Province, and in October 2022 at Nanchang City, Jiangxi Province, China. The field management of the Gansu experimental field followed standard agricultural practices. Each line or parental accession was grown in two-row plots, with about 20 plants in each row. The distance between the two rows of the test site was 30 cm, and the distance between the two adjacent plants was about 20 cm. The RIL population in Nanchang was planted in pots with diameters of 19 cm with 4–5 plants per pot.

### 4.2. LPA and LIA Evaluation

Three representative plants from each line were selected for trait measurement. The third and fourth leaves (from bottom to top) of the plant were photographed at the seedling stage (eight-leaf stage to ten-leaf stage), and the LPA and LIA were calculated using Image J software (https://imagej.net/downloads, accessed on 20 January 2023). The LPA was measured by half of the angle between the two petioles during the measurement process, and the LIA was measured by half of the inclination angle of the two leaves during the measurement process (Supplementary Figure S6).

### 4.3. QTL Mapping

JoinMap 4.0 was used to construct a genetic linkage map. The parameters of the software were set as the fitting threshold ≤5.0, the maximum recombination rate <0.4, and the minimum logarithm of odds (LOD) scores of 3.0 [48]. QTL mapping was performed using QTL IciMapping 4.2 and detected by inclusive composite interval mapping (ICIM). The nomenclature of QTL was similar to that described by Chen et al. with minor modifications [48]. Briefly, a QTL was assigned a lowercase q as the starting point; this was followed by the uppercase three-letter nomenclature of the character name (LPA/LIA); the letter of a capitalized chromosome (A or C), a chromosome number, a point, and a lowercase letter (a, b, c) were added to multiple QTLs detected on the same linkage group and environment to distinguish between them [48].

### 4.4. Transcriptome Analysis

Two lines from the RIL population with significant differences in LPA, ‘R036’ and ‘R009’, were selected for RNA-seq. The main stem and petiole of the fourth leaf (from bottom to top) of ‘R036’ and ‘R009’ at the nine-leaf stage were sampled for RNA-seq. Fastp software (https://github.com/OpenGene/fastp (accessed on 4 January 2024)) was used to filter the raw reads. The filtering principle was as follows: remove the reads containing adaptors; remove reads with more than 10% N ratio; and remove low-quality (mass value of less than 20) bases accounting for more than 50% of the reads. Before sequence alignment, the reference genome file of Zhongshuang 11 was downloaded from Ensembl Plants (http://plants.ensembl.org (accessed on 6 January 2024)) [49]. The qualifying data were compared with the reference genome using hisat2 software (https://daehwankimlab.github.io/hisat2/ (accessed on 6 January 2024)) [50]. DEGs were defined as |FoldChange| ≥ 1 and *p*-adjust (FDR) ≤ 0.05. TBtools was used for Gene Ontology (GO) enrichment analysis, and results with *p*-adjusted (FDR) < 0.05 were significantly enriched. The GO term satisfying this condition was defined as the GO term that was significantly enriched in DEGs.

### 4.5. Anatomical Structure of Stem-Petiole Junction 

Two lines from the RIL population with significant differences in LPA, ‘R036’ and ‘R009’, were sampled for anatomical structure comparison. The connection between the petiole and the stem of the fourth leaf (from bottom to top) at the nine-leaf stage was taken as a sample and fixed with the FAA solution. The samples were sent to the company (Beijing Zhongke Wanbang Biotechnology Co., Ltd. (Beijing, China)) to be cut longitudinally or cross-cut into paraffin sections, which were subsequently observed under an optical microscope (NIKON CI-S, KF-PRO-120).

### 4.6. qPCR Analyses of the Candidate Genes

Two lines from the RIL population with significant differences in LPA, ‘R036’ and ‘R009’, were sampled for RNA extraction. Reverse transcription of RNA was used to obtain single-stranded cDNA (TRUEscript RT Kit +gDNA Eraser, Aidlab, Beijing, China). The primer list of candidate genes is shown in Supplementary Table S5, which was synthesized by Beijing Tsingke Biotech Co., Ltd (Beijing, China). qPCR analysis was performed on a LightCycler^®^ Instrument (Roche) using ChanQ Universal SYBR qPCR Master Mix (Vazyme, Nanjing, China). We used the 2^−ΔΔCt^ method to calculate the expression levels of these five candidate genes [51].

## 5. Conclusions

Overall, genetic, molecular, and cytological results collectively showed that cell wall activity and the biosynthesis/metabolism of cell wall components played an important role in regulating the LA trait in rapeseed. This study has provided a good basis for the improvement of LA traits and high-density planting of rapeseed.

## Figures and Tables

**Table 1:** LPA and LIA for the two parents and derived RIL population in the two investigated environments. SD: standard deviation CV: coefficient of variation LPA: leaf petiole angle LIA: leaf inclination angle. *: *p* < 0.05 significant difference.

Environment	Materials		LPA	LIA
Gansu	Parents	M201	29.75° ± 0.095	50.30° ± 1.20
		M352	54.89° ± 1.36	68.07° ± 0.90
		*Pt*-test	0.016 *	0.018 *
	RIL	Min	22.73°	29.68°
		Max	61.69°	81.04°
		Mean ± SD	41.61° ± 8.26	55.79° ± 10.43
		CV	19.85%	18.70%
Nanchang	Parents	M201	25.75° ± 0.49	34.70° ± 0.22
		M352	34.76° ± 0.52	45.39° ± 1.20
		*Pt*-test	0.036 *	0.020 *
	RIL	Min	18.44°	21.31°
		Max	52.82°	64.07°
		Mean ± SD	31.16° ± 6.98	42.31° ± 8.52
		CV	22.40%	20.14%

**Table 2:** QTL mapping results of LPA and LIA.

Trait	QTL	Chromosome	Confidence Interval	LOD	PVE (%)	Add (%)	Environment	Physical Location
LPA	*qLPAA01*	A01	52.5–66.5	2.5872	7.7860	−2.5216	Gansu	6,239,139–9,453,887
	*qLPAA10.a*	A10	39.5–40.5	2.8796	6.7048	2.3380	Gansu	18,971,020–19,039,409
	*qLPAA10.b*	A10	28.5–33.5	2.6689	0.4409	−1.8276	Nanchang	18,091,839–18,522,230
	*qLPAC03*	C03	107.5–108.5	37.4487	11.2860	9.2192	Nanchang	18,838,385–19,430,815
LIA	*qLIAA06*	A06	17.5–19.5	3.1420	0.9141	2.3155	Nanchang	3,105,210–3,152,990
	*qLIAC03.a*	C03	107.5–108.5	34.1515	17.4869	10.0803	Nanchang	18,838,385–19,430,815
	*qLIAC03.b*	C03	144.5–150.5	2.8696	0.8706	−2.2593	Nanchang	7,166,121–8,185,264

**Table 3:** Cell size of the connection part between the petiole and stem.

No.	Cell Diameter (μm)	Cell Surface Area (μm^2^)
R009	19.13 ± 6.33	200.82 ± 114.40
R036	11.79 ± 3.14	74.20 ± 38.68
*p*-value	4.01 × 10^−5^	3.49 × 10^−5^

**Table 4:** Candidate genes for QTL and RNA-seq combined analysis.

QTL	Rapeseed Gene	Arabidopsis Gene	Gene Function
*qLPAA01*	*BnaA01G0125600ZS*	*AT4G22680*	Encodes a putative transcription factor
	*BnaA01G0135700ZS*	*AT4G24000*	Cellulose synthase-like protein G2
	*BnaA01G0154600ZS*	*AT4G25960*	P-glycoprotein 2
*qLPAA10.a*	*BnaA10G0154200ZS*	*AT5G60020*	*Laccase-17*
*qLPAC03*	*BnaC03G0294200ZS*	*AT4G08770*	peroxidase Prx37

## Data Availability

The original contributions presented in the study are included in the article/Supplementary Materials, further inquiries can be directed to the corresponding authors.

## Supplementary Materials

The following supporting information can be downloaded at: https://www.mdpi.com/article/10.3390/ijms25179325/s1.
