# Supplementary material for: Mining Candidate Genes for Leaf Angle in Brassica napus L. by Combining QTL Mapping and RNA Sequencing Analysis
Source: Int J Mol Sci. 2024 Aug 28;25(17):9325. doi: 10.3390/ijms25179325 (PMC11394825; doi:10.3390/ijms25179325)
Supplement: Supplementary file 1 [file ijms-25-09325-s001.zip › Supplementary Materials.pdf]

Supplementary Table S1. Genes contained in QTL mapping interval.

Supplementary Table S2. Sequence alignment between the sequenced sample and the reference genome.

Sample	Raw reads	Clean reads	Q30 rate (%)	GC content (%)	Total mapped	Uniquely mapped
S1	54007116	54007108	96.51	46.22	50402100 (93.32%)	47304693 (87.59%)
S2	56329448	56329434	96.51	46.38	52615187 (93.41%)	49233759(87.40%)
S3	63370614	63370600	96.95	46.00	58848113 (92.86%)	54988922 (86.77%)
B1	67709798	67709790	96.65	46.16	63090274 (93.18%)	59119442 (87.31%)
B2	58641492	58641476	96.65	46.04	54777296 (93.41%)	51497571 (87.82%)
B3	76798078	76798048	96.71	45.89	70940101 (92.37%)	66368930 (86.42%)

Supplementary Table S3. Analysis of KEGG enrichment of the DEGs.

Supplementary Table S4. DEGs contained in the QTL mapping interval.

Supplementary Table S5. Primers for candidate genes.

Rapeseed gene	Primer name	Primer sequence (5' to 3')
BnaA01G0125600ZS	1F	CCCAAAAACATTTCCTCGACTTCA
	1R	AGTTTCGTCGGACCATAAGTGG
BnaA01G0135700ZS	2F	GGAACTGTGAATGTAGGCGTAC
	2R	AGGTTTCTTCTGCTCCACTGG
BnaA01G0154600ZS	3F	GTGAGATTGCGGAAGAGGTG
	3R	GACAACAACGCTTGTGAACCA
BnaA10G0154200ZS	4F	CATTCAGACTGAGAGTGAAGCC
	4R	TCAGAAGCAGGTTTGTGGTCT
BnaC03G0294200ZS	5F	TGTCACTTTGGCAGGAGGTC
	5R	CCAAAAGTGTGACCACCAGAG
	actinQ3-F	CTATCCTCCGTCTCGATCTCGC
	ActinQ3-R	CTTAGCCGTCTCCAGCTCTTGC

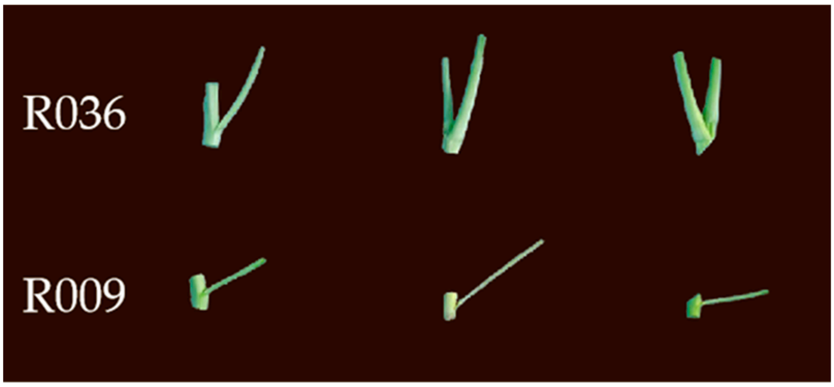

Supplementary Figure S1. LPA comparison of R036 and R009.

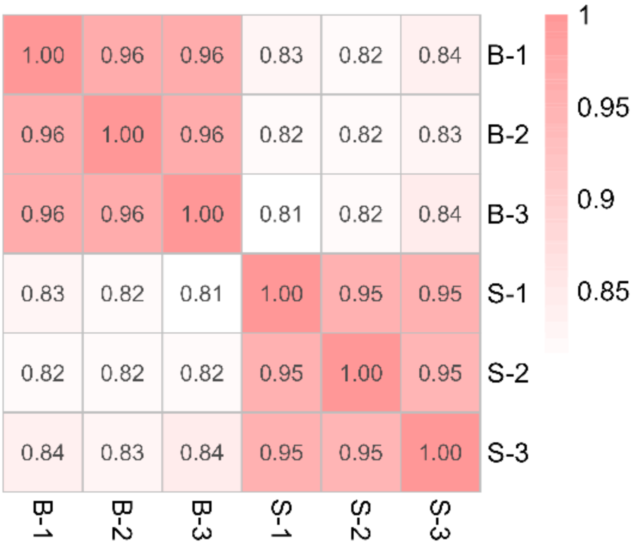

Supplementary Figure S2. Correlation analysis of B and S. S: R036 B: R009.

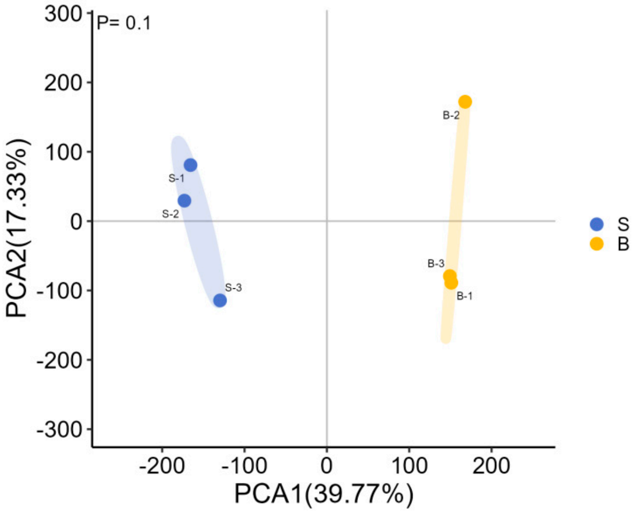

Supplementary Figure S3. Principal component analysis of R036 and R009.　S：R036 B：R009.

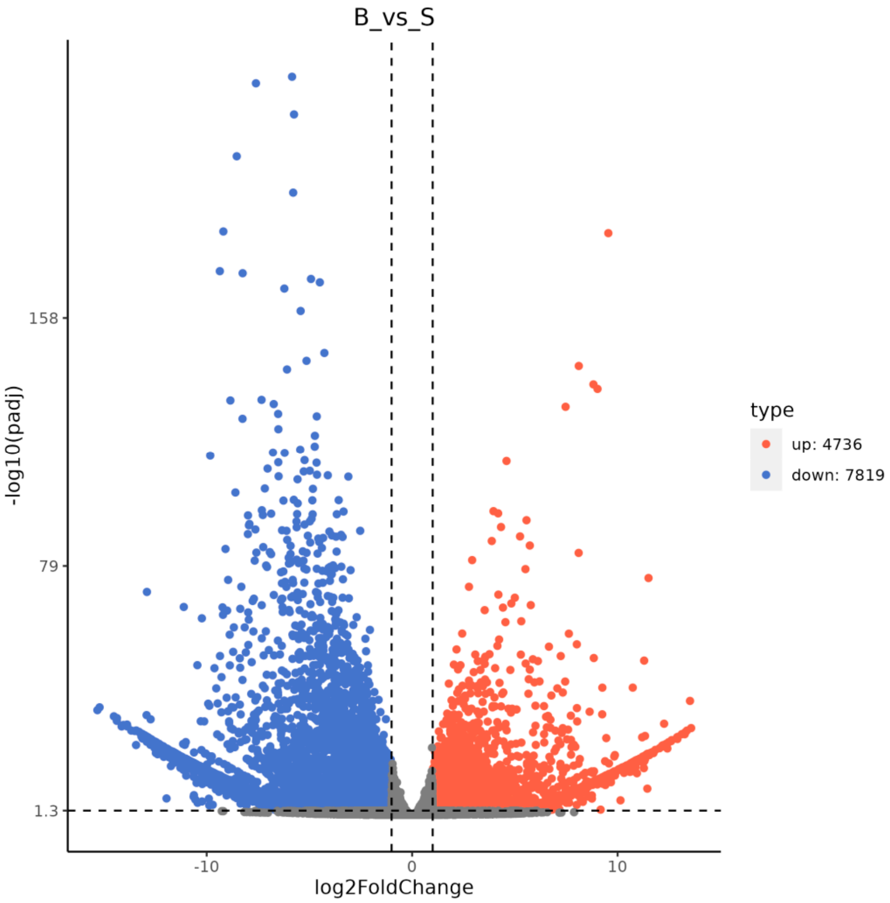

Supplementary Figure S4. DEGs volcano map. S: R036 B: R009.

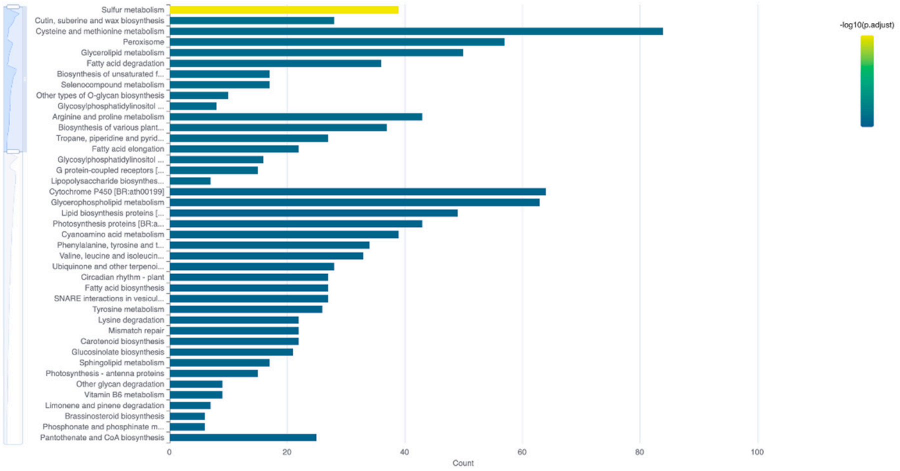

Supplementary Figure S5. KEGG enrichment analysis of DEGs between R036 and R009.

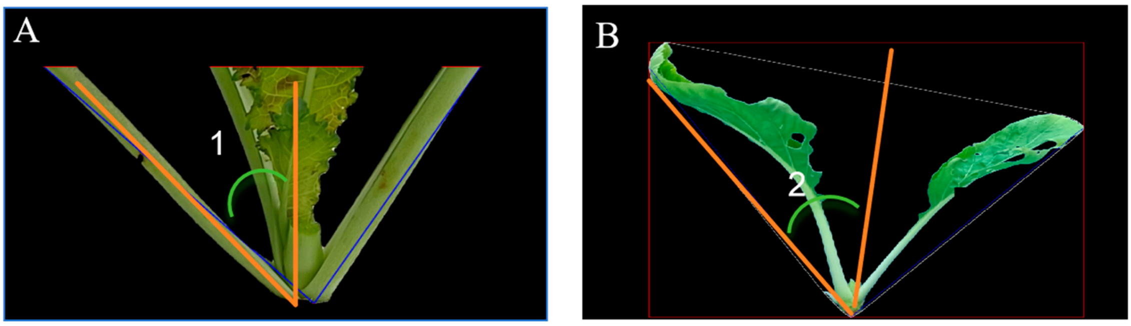

Supplementary Figure S6. Leaf angle diagram. (A) ∠1: leaf petiole angle (LPA). (B) ∠2: leaf inclination angle (LIA).
